# Response durability after cessation of immune checkpoint inhibitors in patients with metastatic Merkel cell carcinoma: a retrospective multicenter DeCOG study

**DOI:** 10.1007/s00262-021-02925-4

**Published:** 2021-04-18

**Authors:** H. M. Stege, M. Haist, S. Schultheis, M. I. Fleischer, P. Mohr, S. Ugurel, P. Terheyden, A. Thiem, F. Kiecker, U. Leiter, J. C. Becker, M. Meissner, J. Kleeman, C. Pföhler, J. Hassel, S. Grabbe, C. Loquai

**Affiliations:** 1 Department of Dermatology, University Medical Center of the Johannes Gutenberg University, Mainz, Germany; 2 Department of Dermatology, Elbe-Kliniken Buxtehude, Buxtehude, Germany; 3 Department of Dermatology, University Medical Center Essen, Essen, Germany; 4 Department of Dermatology, Allergology and Venerology, University Lübeck, Lübeck, Germany; 5 Department of Dermatology, University Medical Center Würzburg, Würzburg, Germany; 6 Department of Dermatology, University Medical Center Rostock, Rostock, Germany; 7 Department of Dermatology and Allergology at the Charité, University Medical Center Berlin, Berlin, Germany; 8 Dermato-Oncology, Department of Dermatology, University Medical Center Tübingen, Tübingen, Germany; 9 German Consortium for Translational Oncology (DKTK) and German Cancer Research Center (DKFZ), Heidelberg, Germany; 10 Department of Dermatology, University Medical Center Frankfurt, Frankfurt, Germany; 11 Department of Dermatology, University Medical Center Homburg, Homburg, Germany; 12 Department of Dermatology, University Medical Center Heidelberg, Heidelberg, Germany

**Keywords:** Immune checkpoint inhibitors, Metastatic Merkel cell carcinoma, Duration of response, Treatment cessation, Rechallenge of immune checkpoint inhibitors

## Abstract

**Background:**

Immune checkpoint inhibitors (ICI) have led to a prolongation of progression-free and overall survival in patients with metastatic Merkel cell carcinoma (MCC). However, immune-mediated adverse events due to ICI therapy are common and often lead to treatment discontinuation. The response duration after cessation of ICI treatment is unknown. Hence, this study aimed to investigate the time to relapse after discontinuation of ICI in MCC patients.

**Methods:**

We analyzed 20 patients with metastatic MCC who have been retrospectively enrolled at eleven skin cancer centers in Germany. These patients have received ICI therapy and showed as best overall response (BOR) at least a stable disease (SD) upon ICI therapy. All patients have discontinued ICI therapy for other reasons than disease progression. Data on treatment duration, tumor response, treatment cessation, response durability, and tumor relapse were recorded.

**Results:**

Overall, 12 of 20 patients (60%) with MCC relapsed after discontinuation of ICI. The median response durability was 10.0 months. Complete response (CR) as BOR to ICI-treatment was observed in six patients, partial response (PR) in eleven, and SD in three patients. Disease progression was less frequent in patients with CR (2/6 patients relapsed) as compared to patients with PR (7/11) and SD (3/3), albeit the effect of initial BOR on the response durability was below statistical significance. The median duration of ICI therapy was 10.0 months. Our results did not show a correlation between treatment duration and the risk of relapse after treatment withdrawal. Major reasons for discontinuation of ICI therapy were CR (20%), adverse events (35%), fatigue (20%), or patient decision (25%). Discontinuation of ICI due to adverse events resulted in progressive disease (PD) in 71% of patients regardless of the initial response. A re-induction of ICI was initiated in 8 patients upon tumor progression. We observed a renewed tumor response in 4 of these 8 patients. Notably, all 4 patients showed an initial BOR of at least PR.

**Conclusion:**

Our results from this contemporary cohort of patients with metastatic MCC indicate that MCC patients are at higher risk of relapse after discontinuation of ICI as compared to melanoma patients. Notably, the risk of disease progression after discontinuation of ICI treatment is lower in patients with initial CR (33%) as compared to patients with initial PR (66%) or SD (100%). Upon tumor progression, re-induction of ICI is a feasible option. Our data suggest that the BOR to initial ICI therapy might be a potential predictive clinical marker for a successful re-induction.

## Introduction

Immune checkpoint inhibitors (ICI) changed the treatment landscape for patients with advanced MCC. Prior to the introduction of this class of medication, chemotherapy represented the only treatment option for patients with metastatic MCC. In general, chemotherapy generates a good initial response, but often does not result in a significant survival benefit, since patients rapidly develop chemotherapy resistance and relapse. In light of the good response to ICI in immunogenic tumor entities such as malignant melanoma, single-arm clinical trials investigating the efficacy of ICI in metastatic MCC were encouraged. These studies did not only show a good initial response, but also a significantly prolonged progression-free (PFS) and overall survival (OS) for patients allocated to ICI therapy [1–3]. The safety profiles of the anti-programmed-cell death protein (PD)-1 and anti-programmed-cell death-protein–ligand (PD-L1) antibodies administered to patients with MCC appear similar to those observed in previous trials involving patients with other tumor entities [4]. Recently published data show that ICI protocols are generally well tolerated. Nonetheless, the frequently observed occurrence of immune-related adverse events (irAE) represents one of the main reasons for treatment discontinuation (10–20%) [5]. The clinical course of patients with a favorable initial response, but early treatment termination due to adverse events or personal preference is unknown.

In this study, we aimed to assess outcomes of patients with metastatic MCC after discontinuation of ICI treatment following an initial response to ICI therapy.

## Methods

This study was designed as a retrospective multicenter analysis. The documentation of the retrospective data was carried out using anonymized forms of patients with metastatic MCC who were treated with PD-1-inhibitors/PD-L1-inhibitors at the participating skin cancer centers from March 2015 to December 2019. The participating centers received the anonymized forms in order to document the clinical data of patients who met the inclusion criteria. Documentation of the patients’ medical records was extracted either by medical doctors or by clinical research documentation professionals, depending on the site and merged into a central database prior to analysis. A follow-up period of at least 3 months was required. A total of 20 patients with stage IV, histologically confirmed MCC were enrolled at 35 Skin Cancer Centers in Germany (31), Austria (2), and Switzerland (2). Key eligibility criteria were inoperability of MCC and premature discontinuation of PD-1-inhibitors/PD-L1-inhibitors after initial tumor response to ICI. The multicenter collection of patient data was approved by the Ethics Committee of Rhineland Palatinate (2019-14436) and was conducted in accordance with the principles of the Helsinki Declaration in its current version. Data on the age at the start of PD-1 treatment, sex, primary localization of the MCC, site of metastasis, LDH status in serum, systemic pretreatments, modality of anti-PD-1/anti-PD-L1 treatment (Pembrolizumab, Nivolumab or Avelumab), number of treatment cycles, best response to treatment as assessed via complete response (CR), partial response (PR), stable disease (SD) or progressive disease (PD), response durability, overall status of the patients, the reason for discontinuation, and status after discontinuation of ICI treatment were collected. Due to laboratory abnormalities, time constraints, and immune-related adverse events (irAE) (CTCAE grade 1–2) the recommended therapy intervals for Pembrolizumab (every 3 weeks) and Avelumab (every 2 weeks) could not always be complied. After cessation of anti-PD-1/anti-PD-L1 treatment, all patients were observed via radiological staging and clinical review.

## Statistical analysis

Descriptive statistics were used to assess the baseline characteristics of the study population. Treatment duration was calculated as the period between initial drug administration and the last treatment date before discontinuation of ICI. Response durability was calculated from the date of the last administration cycle of ICI treatment until the date of PD or last follow-up. Kruskal–Wallis rank sum test was applied to determine the correlation between the initial response to ICI and the following duration of response. Logistic regression analysis and Fisher’s exact test were used to identify predictive biomarkers for response durability. As a time-to-event endpoint, this retrospective cohort study used the response durability after discontinuation of ICI, which was estimated using the Kaplan–Meier product-limit method and log-rank statistics in R. SPSS (Version v 23, IBM IBM Ehningen, Germany), RStudio (Version 1.3.959), and GraphPad PRISM (Version 5, GraphPad Software, San Diego, CA, USA) used for all analyses.

## Results

### Baseline patient characteristics and tumor response

From 2015 to 2019, a total of 20 patients (nine female, eleven male) were enrolled in this retrospective study. All patients were diagnosed with an inoperable or metastatic MCC prior to treatment initiation. The mean age at anti-PD-1/anti-PD-L1 treatment initiation was 72.9 years (range 53–97 yrs). The most frequent metastasis locations were lymph nodes (80%) and skin (45%). Twelve patients received the anti-PD-1 antibody Pembrolizumab, seven patients received the anti-PD-L1 antibody Avelumab, and one patient received Nivolumab (Table 1). Prior to the initial application of checkpoint inhibitor treatment, lactate dehydrogenase (LDH) serum levels were elevated in eleven patients (61.1%). Cox regression analyses revealed no significant impact of gender, age, the presence of lymph node metastasis, treatment duration, LDH levels, or systemic pretreatments on the progression-free survival of the investigated patient cohort.

**Table 1:** Clinicopathological characteristics of the investigated cohort discontinuing ICI therapy

Clinicopathological features	*N* (%)
*Median age* at initiation of ICI (range)	73 (53–97)
*Gender*	
Female	9/20 (45%)
Male	11/20 (55%)
*Primary tumor*	
Localization	
CUP^1^	7/20 (35%)
Upper limb	5/20 (25%)
Lower limb	5/20 (25%)
Head–neck area	2/20 (10%)
Trunk	1/20 (5%)
*Positive MCPyV*^2^* status*	4/7 (57%)^4^
*Metastatic lesion*	
Metastatic sites	
Lung	3/20 (15%)
Liver	4/20 (20%)
Nodal	16/20 (80%)
Cutaneous	9/20 (45%)
Cerebral	0/20
Other (Bone, muscles, pancreas, adrenal glands)	7/20 (35%)
*Elevated LDH serum levels at beginning of ICI*	11/18 (61.1%)^5^
*ECOG*^3^*-status ≥ 1 at beginning of ICI*	9/20 (45%)
*Pre-existing immunosuppression (HIV, transplantation, medication)*	0/20
*Treatments*	
Previous treatments (conventional chemotherapy)	9/20 (45%)
Treatment with Checkpoint-inhibitors (first-line)	
Pembrolizumab	12/20 (60%)
Nivolumab	1/20 (5%)
Avelumab	7/20 (35%)
Median treatment duration (range)	10.0 months (3–27 months)
Median response durability (range)	10.0 months (1–24 months)
Treatment-related adverse events	9/20 (45%)
Initiation of immunosuppressive treatment	4/20 (20%)
Discontinuation of ICI-treatment	7/20 (35%)
Progress after ICI discontinuation	12/20 (60%)
Rechallenge of ICI	8/12 (66.7%)
Median time interval between ICI discontinuation and re-induction (range)	7.0 months (1–20 months)
Median response durability upon ICI rechallenge (range)	6.0 months (1–18 months)
*Follow-up*	
Median follow-up period upon ICI discontinuation	13.2 months
Overall observation period, median (range)	20.5 months (4–37 months)
Deceased	5/20 (25%)

^1^*CUP* cancer of unknown primary, ^2^*MCPyV* Merkel cell polyomavirus, ^3^*ECOG* Eastern Cooperative Oncology Group; Percentages based on the total number of patients with known MCPyV and LDH-serum levels

**Fig. 1:**
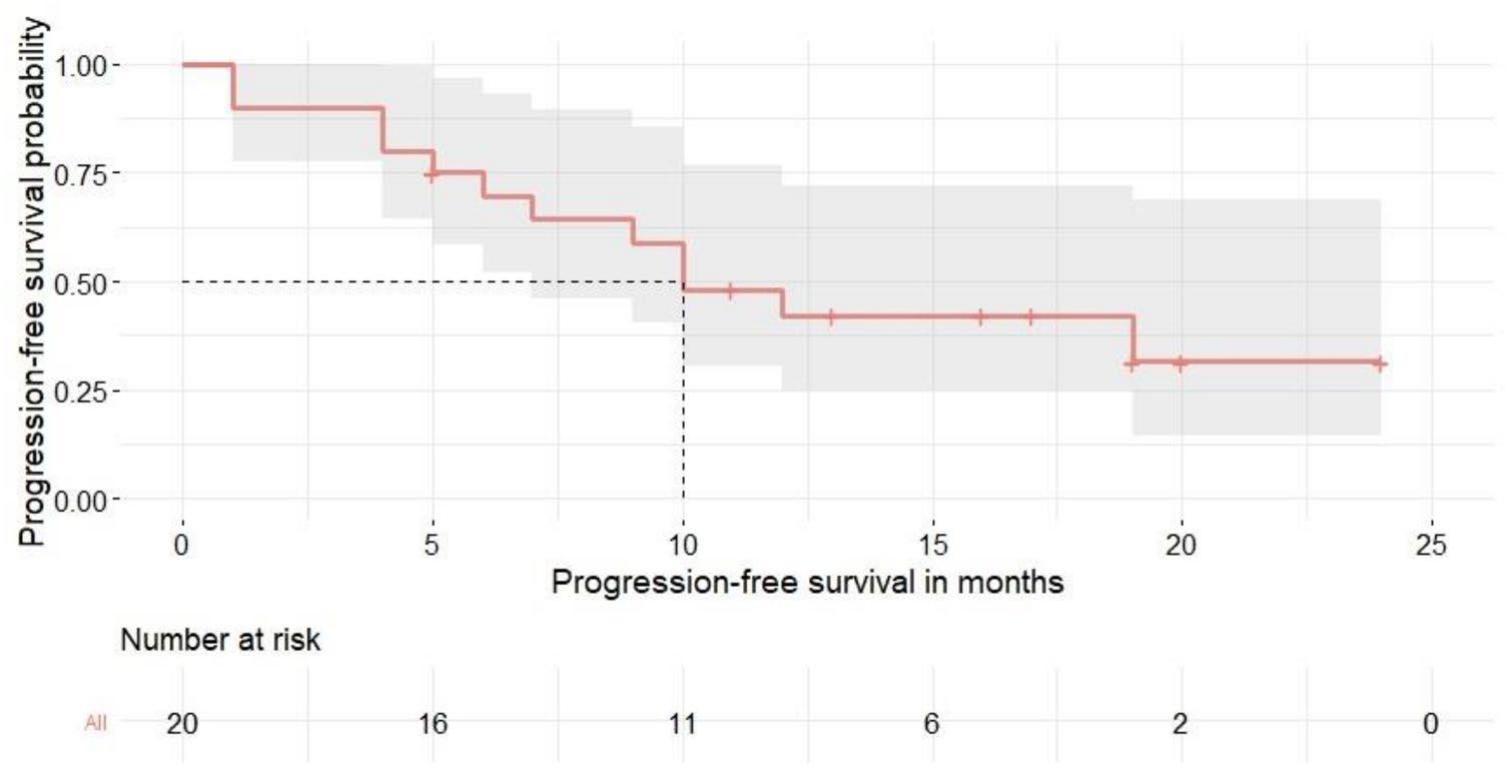
Median progression free survival in patients with advanced MCC. The Kaplan–Meier plot illustrates the median response durability after cessation of ICI therapy (dashed line; median: 10.0 months) and the corresponding 95% confidence interval (gray overlay) of the entire cohort (*n* = 20)

**Fig. 2:**
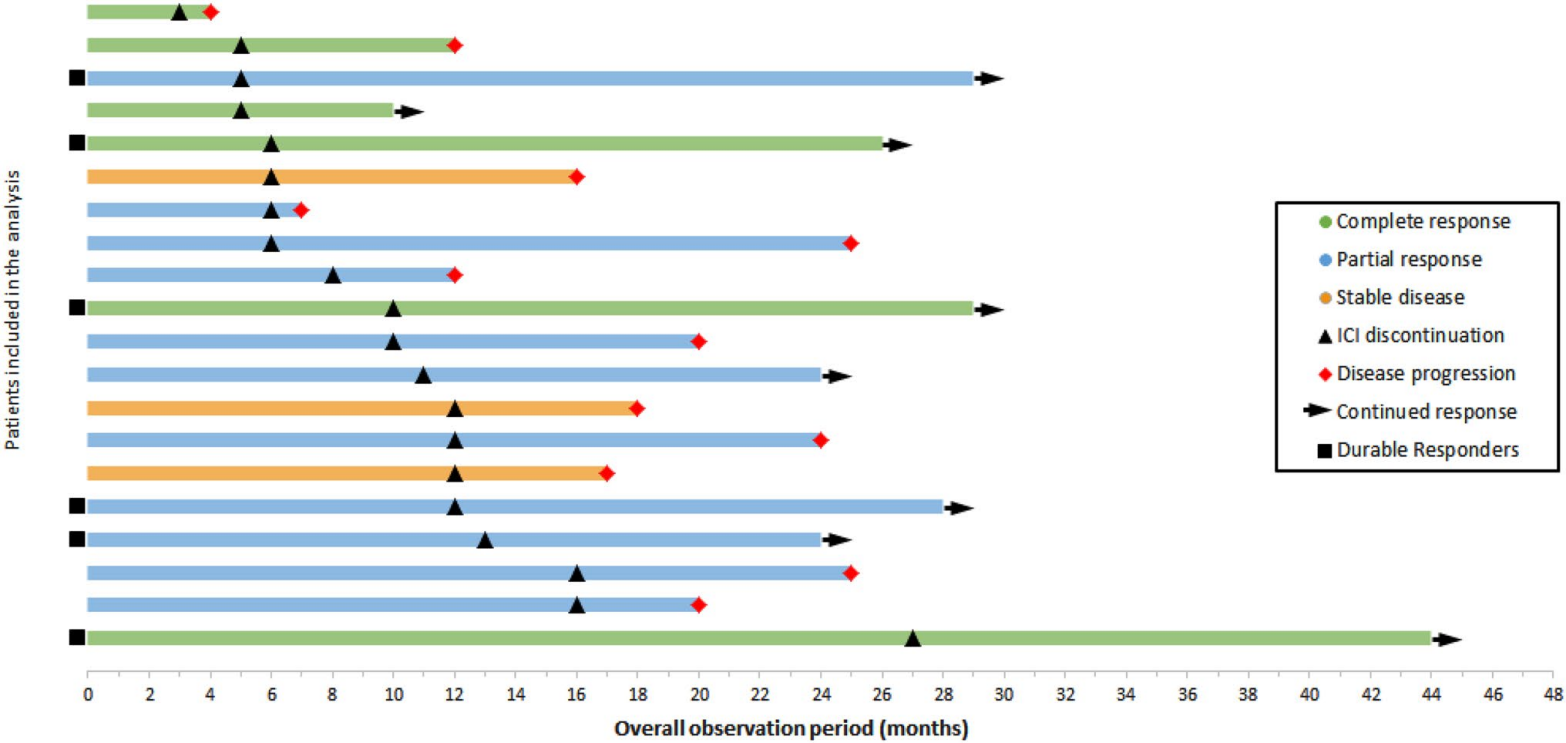
Swimmer’s plot for the patient cohort investigated in the retrospective analysis. Six patients achieved an initial CR to ICI treatment (green bars), 11 patients showed a partial response to ICI therapy (blue bars) and 3 patients showed a stable disease upon ICI treatment (orange bars). After ICI discontinuation we observed disease progression in 12 patients, whereas 8 patients retained an ongoing response

The mean duration of anti-PD-1/anti-PD-L1 treatment was 10.1 months (range 3–27 months) with a median response durability of 10.0 months (95% CI 6.0–14.0 months, as calculated by Kaplan–Meier estimate, Fig. 1) with a median follow-up period of 13.2 months (95% CI: 4.5–19.3 months) Complete response as BOR to the anti-PD-1/anti-PD-L1 treatment was observed in six patients, PR in eleven patients, and SD in three patients (Fig. 2).

The most common causes for premature treatment cessation were irAE (35%). In five patients treatment was discontinued due to patient preference (25%). Four patients discontinued therapy due to overall fatigue or reduced performance status after initial response to ICI therapy (20%). In another four patients treatment was discontinued after complete response to ICI therapy (20%).

### Outcome after ICI discontinuation

Overall, 12 of 20 patients (60%) with MCC relapsed after discontinuation of ICI treatment for reasons other than PD. The median duration of response upon treatment cessation was 10.0 months (95% CI 6.0–14.0 months).

**Fig. 3:**
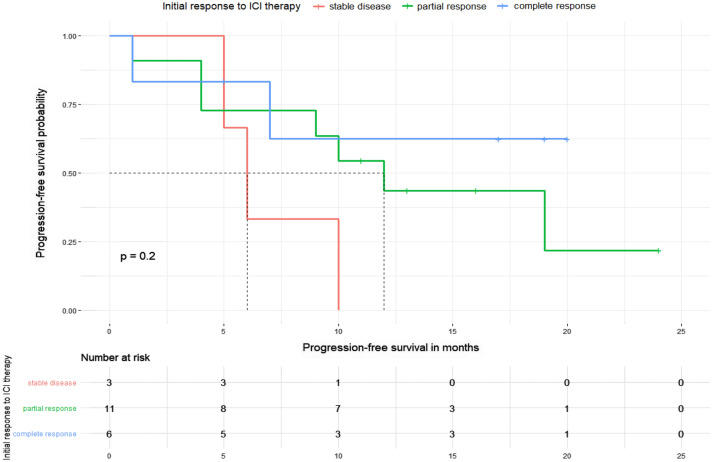
Kaplan–Meier plot illustrating the progression-free survival for the 3 subcohorts of patients showing a different initial response to ICI therapy. It can be found that patients with a SD as BOR (red line) after initiation of ICI therapy have the shortest median PFS (6.0 months) in comparison with those patients showing a PR (green line, median PFS 12.0 months) or a complete response (blue line, median PFS not reached)

The six patients showing an initial CR received ICI for a mean time period of 9.3 months (range 3–27 months). In two (33%) of these patients, treatment was discontinued due to adverse events, while CR was the reason for the discontinuation of anti-PD-1/anti-PD-L1 treatment in 4 (66%) patients. Among patients with an initial CR, an ongoing response was found in 4 patients (66%) with a mean response durability of 11.5 months (1–20 months) at the time of data collection, whereas two patients relapsed with a mean time to relapse of 4 months (range 1–7) (Table 2). Notably, the median progression-free survival, estimated by Kaplan–Meier method, has not been reached in this subcohort of patients (Fig. 3).

**Table 2:** Best overall response and outcome for patients who discontinued ICI treatment

Best overall Response	Number of patients	Patients diagnosed with PD	Mean time of anti-PD-1 treatment	Mean time of response durability after discontinuation
	*N* (%)	*N* (%)	months (range)	months (range)
Complete response	6 (30%)	2 (33%)	9.3 months (range 3–27)	11.5 months (1–20)
Partial response	11 (55%)	7 (66%)	10.4 months (range 5–16)	11.2 months (range 1–24)
Stable disease	3 (15%)	3 (100%)	10 months (range 6–12)	7 months (range 5–10)

ICI, immune checkpoint inhibitors; PD, progressive disease; PD-1, programmed cell death protein 1

Patients showing PR (*n* = 11, Table 2) as the BOR received ICI treatment for an average of 10.4 months (range 5–16 months). The occurrence of grade 3/4 irAE (27%), deterioration of general condition (27%), and patient choice (45%) were among the reasons for discontinuation of ICI treatment. In 63% of these patients, tumor progression was observed within a mean time period of 11.2 months (1–24 months). An ongoing response was found in 4 (36%) patients lasting for a mean time period of 16 months (range 11–24 months). Median progression-free survival in Kaplan–Meier analysis was estimated at 12 months.

Patients with SD as BOR (*n* = 3, Table 2) received ICI therapy for a mean time period of 10 months (range 6–12), while the mean response durability was only 7 months (5–11 months). Median progression-free survival was estimated at 6 months. All three patients with an initial SD later developed a progressive disease. The main reason for cessation of ICI were irAE (*n* = 2/3), which required a subsequent treatment with glucocorticoids (100%).

**Fig. 4:**
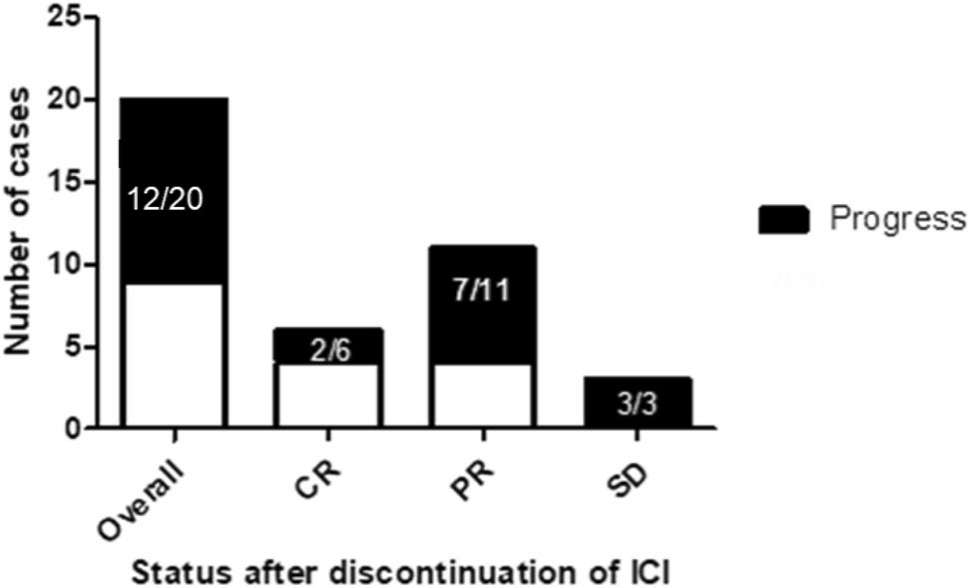
Graphical summary for the number of cases showing tumor progression after termination of ICI-therapy and best response to anti-PD-1/anti-PD-L1 treatment. It can be demonstrated that patients with a strong initial response to anti-PD-1/anti-PD-L1 treatment are more likely to obtain a durable anti-tumor immune response. No progress of metastatic MCC could be found in 36% and 66% of patients, which have shown a partial response or complete response upon ICI treatment. By contrast, disease progression upon treatment cessation was observed among all patients (100%) which had initially shown a stable disease upon ICI treatment. The correlation between the risk of disease progression and the initial BOR was, however, found to be below statistical significance (Fisher´s exact test, *p* = 0.164)

**Fig. 5:**
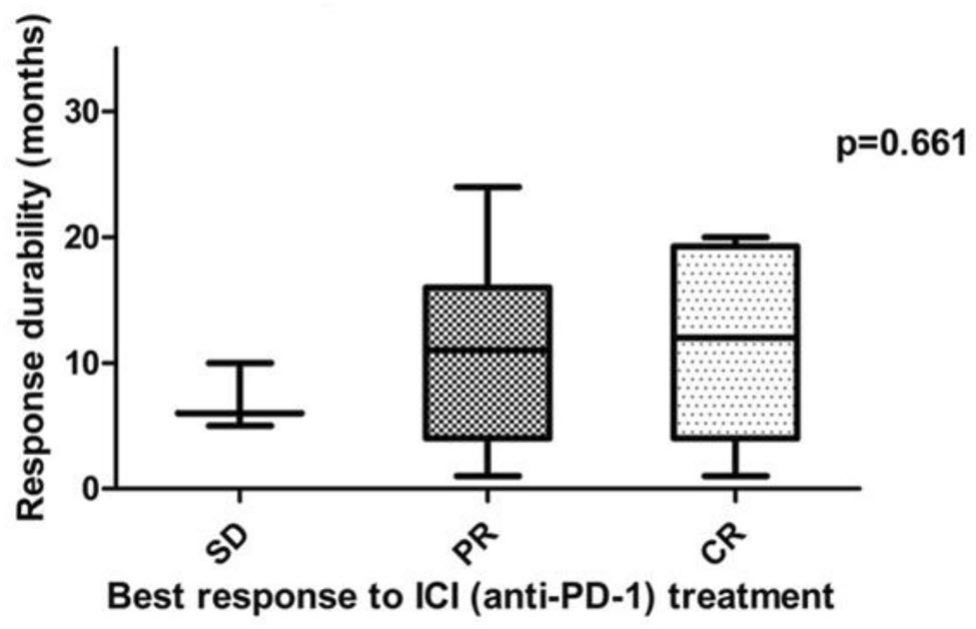
Potential correlation between the response durability and the best response to ICI treatment. It could be demonstrated that the initial response to ICI treatment impacts the long-term response durability (SD: 7 months vs. PR: 11.2 months vs. CR: 11.5 months), albeit this correlation was below statistical significance as analyzed by Kruskal–Wallis rank sum Test (*p* = 0.661)

After discontinuation of ICI therapy, tumor progression could was observed in 12/20 patients. Patients with an ongoing tumor response after discontinuation of ICI showed at least PR as BOR to the initial ICI treatment. By contrast, tumor progression was observed in all patients which showed SD upon ICI treatment (Fig. 4). Hence, our data indicate that the response durability after discontinuation of ICI therapy might be correlated with the initial response to ICI therapy, i.e., patients showing an initial CR towards ICI treatment, having the longest median response durability (see Figs. 3, 5). The duration of initial ICI therapy and the occurrence of severe treatment-related side effects (≥ grade 2, median PFS: 12.0 months vs. < grade 2, median PFS: 9.0 months) were not found to affect the response durability upon treatment discontinuation.

Overall, ICI treatment was discontinued due to adverse events, such as pneumonitis, hepatitis or myocarditis CTCAE ≥ 2, in 7/20 (35%) patients, and in 4 patients the systemic application of corticosteroids (1 mg/kg body weight) was required. Upon obtaining glucocorticoids, 3 of 4 patients experienced progression of the disease. Consistent with this finding, we could observe that most patients (5/7) who had to discontinue ICI therapy, due to severe immune-related adverse events (irAE), subsequently showed a progression of the disease, whereas disease progression was less frequent in patients terminating ICI therapy due to other reasons (7/13). Statistical analysis, using Fisher´s exact test, did, however, not reveal a significantly higher risk of disease progression in patients terminating ICI due to irAE, which might be attributed to the low number of patients investigated (*p* = 0.64).

### Re-induction of ICI upon tumor progression

**Fig. 6:**
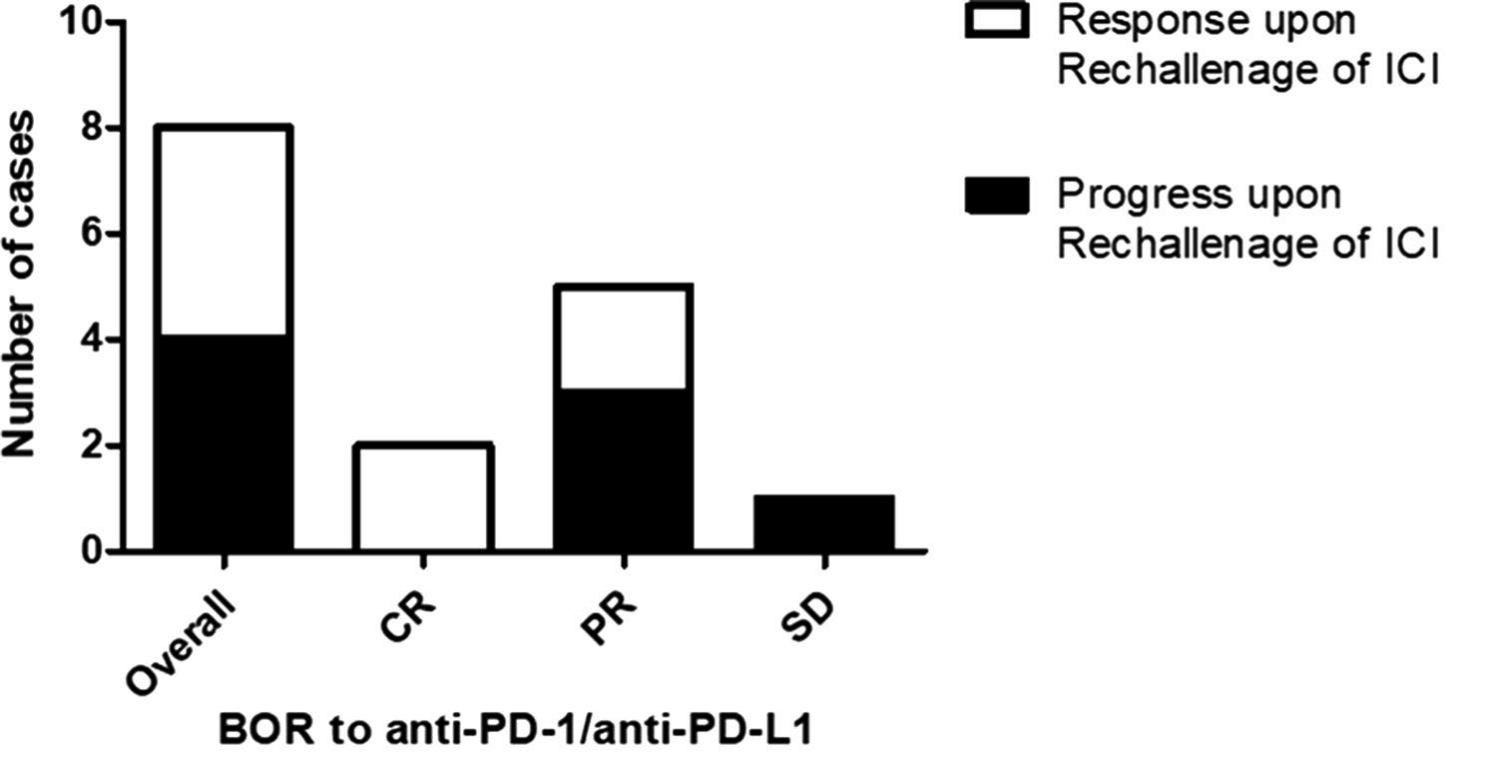
Bar chart illustrating the response to anti-PD-1/anti-PD-L1 re-challange after previous treatment cessation and the best overall response to an initial ICI therapy. Patients with a stronger initial response to ICI therapy are at lower risk of disease progression upon ICI-rechallenge. In particular, it could be found that patients with a CR to initial ICI therapy subsequently achieved at least a partial response upon ICI rechallenge. By contrast, all patients initially showing a SD to ICI therapy progressed upon ICI rechallenge. Patients with an initial PR to ICI therapy showed a heterogeneous outcome after re-induction of ICI therapy (2/5 obtained a response, whereas 3 progressed after re-induction of ICI therapy)

Upon tumor progression, 8 of 12 patients received a re-initiation of ICI therapy. In these patients, a renewed tumor response has been achieved in 4 patients, which showed PR as BOR to the re-induction of ICI. Interestingly, a renewed tumor response could only be observed in patients having achieved at least PR as BOR to the initial ICI treatment. In contrast, all patients with initial SD experienced a tumor progression after the re-induction of ICI (Fig. 6). Results from Cox regression analysis investigating the impact of gender, age, treatment duration and initial response to ICI revealed no significant impact of these characteristics on a successful re-induction of ICI.

## Discussion

The advent of ICI therapy has significantly improved the prognosis of patients with metastatic MCC. Therefore, current guidelines recommend anti-PD-1/anti-PD-L1 treatment as a first-line treatment in patients with metastatic MCC [6, 7]. So far, an optimal duration of ICI therapy has not been established, because discontinuation of ICI therapy occurred in most clinical trials either at the time of disease progression or due to severe adverse events [8, 9]. To determine the outcome of patients after treatment cessation, we investigated the response durability after cessation and re-induction of ICI in a real-world patient cohort with metastatic MCC in this retrospective study.

Severe adverse events during ICI therapy are common and often lead to discontinuation of ICI treatment [2]. However, it remains unknown whether an early discontinuation of ICI therapy in patients with favorable initial response might negatively impact long-term outcomes of patients with MCC. Ongoing response durability after discontinuation of ICI due to severe adverse events has been observed in a substantial proportion of patients with advanced melanoma, a tumor being similarly characterized by a strong immunogenicity [10]. In particular, recent studies reported high rates of a sustained response to ICI therapy in patients with metastatic melanoma if patients had received a minimum 24 months of ICI treatment and have shown a strong initial response to ICI therapy [11, 12]. Moreover, a recent evaluation revealed that melanoma patients treated with a combined ICI therapy (ipilimumab plus nivolumab) did not profit from further immunotherapy beyond the second treatment cycle [13].

In our study, patients with metastatic MCC were treated with ICI for 10.1 months. Compared to the JAVELIN Merkel 200 study [2, 9], patients included in our study were treated considerably longer in a real-life situation. Even though anti-PD-1/anti-PD-L1 treatment is generally well-tolerated, adverse events are still among the main reasons for discontinuation of the ICI [8, 14, 15]. Aligning with these observations, 7 of 20 patients included in our study were withdrawn from therapy due to irAEs.

Cessation of ICI was associated with a significant risk of tumor progression in our cohort of patients with advanced MCC (60%), whereas discontinuation of ICI in advanced melanoma patients does not regularly result in tumor progression [16] and most patients remain relapse-free after the termination of ICI [11, 12, 17–19].

In contrast with previous reports showing a significant correlation between the BOR and the duration of treatment in advanced melanoma patients, we could not observe this correlation in our cohort of patients with advanced MCC [16]. Additionally, our observations revealed that tumor progression occurred at an early time point after discontinuation, which highlights the importance of a frequent follow-up upon treatment cessation. Consistent with this finding, our results suggest that a discontinuation of ICI therapy due to adverse events might require a fast re-induction of therapy in order to prevent a subsequent progression of disease.

Another reason for discontinuation of ICI treatment was the occurrence of a CR (20%). Although the median treatment duration was the shortest in this subgroup (6 months vs. 10 months for PR and SD, respectively), we did not find evidence for an increased risk of disease progression after termination of ICI treatment in patients who initially showed CR. By contrast, patients who initially showed a PR or SD are at higher risk of disease progression after discontinuation of ICI therapy. Similar results have been reported in patients with advanced melanoma, in whom a durable response was more likely for patients with CR upon ICI therapy and a minimum treatment duration of at least 6 months [2, 17].

Furthermore, our data indicate that the re-induction of ICI might be a feasible option following tumor progression after discontinuation of ICI [20]. In this challenging situation, the initial response to ICI treatment may likely serve as a potential predictive marker for the successful re-induction of ICI therapy. We could observe a renewed response to ICI in 50% of our patients. Notably, response has been limited to patients with an initial PR to ICI. Similar response rates to a re-induction of ICI have been previously reported for other immunogenic tumor entities such as malignant melanoma [21–23].

The retrospective design and the small sample size are significant limitations of this study. Therefore, a larger, prospective cohort study is needed to validate the observations from our study and confirm the potential value of the initial BOR in the prediction of the response durability toward ICI treatment. Due to the administration of the treatment by multiple centers, there may have been further variabilities in terms of the timing of staging, although staging was typically performed every 3 months after treatment.

In summary, our results indicate that durable responses to ICI therapy can be achieved best in patients with a CR as initial response toward ICI. In contrast with previous observations made for patients who have discontinued ICI therapy in the metastatic melanoma setting [24], our results do, however, suggest that patients with metastatic MCC and an initial complete response to ICI therapy might be at a higher risk of disease progression as compared to melanoma patients. Therefore, we propose that cessation of ICI therapy should be as short as possible with a need for timely re-initiation of treatment, if treatment associated irAE should force a discontinuation of ICI treatment. The prompt re-induction of ICI therapy might be the most feasible option for MCC patients with an initial PR to ICI therapy. Last, our observations highlight the necessity for further investigations defining (i) potential clinical markers predicting response durability both after initiation and after discontinuation of ICI therapy and (ii) the duration of ICI treatment to achieve a sustained tumor control.

## Data Availability

The data and material are available as described in the methods and within this publication and its supplementary material. Statistical analysis was performed using SPSS (Version v 23, IBM IBM Ehningen, Germany), RStudio (Version 1.3.959), and GraphPad PRISM (Version 5, GraphPad Software, San Diego, CA, USA).
